# Short-Chain Fatty Acids Ameliorate Diabetic Nephropathy via GPR43-Mediated Inhibition of Oxidative Stress and NF-*κ*B Signaling

**DOI:** 10.1155/2020/4074832

**Published:** 2020-08-01

**Authors:** Wei Huang, Yi Man, Chenlin Gao, Luping Zhou, Junling Gu, Huiwen Xu, Qin Wan, Yang Long, Li Chai, Youhua Xu, Yong Xu

**Affiliations:** ^1^Department of Endocrinology, Affiliated Hospital of Southwest Medical University, Luzhou, Sichuan, China 646000; ^2^Faculty of Chinese Medicine, Macau University of Science and Technology, Avenida Wai Long, Taipa, Macau, China; ^3^State Key Laboratory of Quality Research in Chinese Medicine, Macau University of Science and Technology, Avenida Wai Long, Taipa, Macau, China; ^4^Luzhou Key Laboratory of Cardiovascular and Metabolic Diseases, Luzhou, Sichuan, China 646000; ^5^Key Laboratory of Medical Electrophysiology of Ministry of Education, Collaborative Innovation Center for Prevention and Treatment of Cardiovascular Disease of Sichuan Province, Southwest Medical University, Luzhou, Sichuan 646000, China; ^6^Sichuan Clinical Research Center for Nephropathy, Luzhou, Sichuan, China 646000; ^7^Affiliated Hospital of Southwest Medical University, Luzhou, Luzhou, Sichuan, China 646000; ^8^Department of Pathology, Affiliated Hospital of Southwest Medical University, Luzhou, Sichuan, China 646000

## Abstract

Diabetic nephropathy (DN) is a chronic low-grade inflammatory disease. Oxidative stress and nuclear factor kappa B (NF-*κ*B) signaling play an important role in the pathogenesis of DN. Short-chain fatty acids (SCFAs) produced from carbohydrate fermentation in the gastrointestinal tract exert positive regulatory effects on inflammation and kidney injuries. However, it is unclear whether SCFAs can prevent and ameliorate DN. In the present study, we evaluated the role and mechanism of the three main SCFAs (acetate, propionate, and butyrate) in high-fat diet (HFD) and streptozotocin- (STZ-) induced type2 diabetes (T2D) and DN mouse models and in high glucose-induced mouse glomerular mesangial cells (GMCs), to explore novel therapeutic strategies and molecular targets for DN. We found that exogenous SCFAs, especially butyrate, improved hyperglycemia and insulin resistance; prevented the formation of proteinuria and an increase in serum creatinine, urea nitrogen, and cystatin C; inhibited mesangial matrix accumulation and renal fibrosis; and blocked NF-*κ*B activation in mice. SCFAs also inhibited high glucose-induced oxidative stress and NF-*κ*B activation and enhanced the interaction between *β*-arrestin-2 and I-*κ*B*α* in GMCs. Specifically, the beneficial effects of SCFAs were significantly facilitated by the overexpression GPR43 or imitated by a GPR43 agonist but were inhibited by siRNA-GPR43 in GMCs. These results support the conclusion that SCFAs, especially butyrate, partially improve T2D-induced kidney injury via GPR43-mediated inhibition of oxidative stress and NF-*κ*B signaling, suggesting SCFAs may be potential therapeutic agents in the prevention and treatment of DN.

## 1. Introduction

Diabetic nephropathy (DN) is a serious microvascular complication of diabetes and a major cause of end-stage renal disease (ESRD) [1]. Oxidative stress, NF-*κ*B signaling activation, and overexpression of various inflammatory cytokines caused by a persistent hyperglycemic state as well as hemodynamic changes play an important role in the pathogenesis of DN. These events cause glomerulosclerosis, tubular atrophy, and fibrosis, eventually leading to irreversible renal damage [2]. However, as clinical strategies based on antioxidant stress and anti-inflammation are limited in their effectiveness, it is becoming increasingly important to explore new methods for the prevention and treatment of DN [3].

The gut microbiota and its metabolites play pivotal roles in host physiology and pathology [4]. Short-chain fatty acids (SCFAs), mainly acetate, propionate, and butyrate, with their ratio in the colon being 60 : 25 : 15, respectively, are produced predominantly by gut microbiota in the fermentation of dietary fiber and undigested carbohydrates [5, 6]. SCFAs are involved in the maintenance of a gastrointestinal epithelial barrier, the regulation of hormone secretion, and the inhibition of enteric endotoxemia and inflammation via the combination of a cell surface G-protein-coupled receptor (GPCR) pair of GPR41 and GPR43 or by inhibiting histone deacetylation (HDAC) [7, 8]. In clinical investigations and animal models, the increased intake of dietary fibers or SCFA administration has also been shown to possess protective effects in inflammatory bowel conditions, allergic airway disease, obesity, type 1 diabetes (T1D), and type 2 diabetes (T2D) due to their inhibitory effects on proinflammatory cytokines and reactive oxygen species (ROS) [9, 10]. These studies show oxidative stress and NF-*κ*B signaling as effector mechanisms of SCFAs, suggesting a promising therapeutic potential in the treatment of chronic low-grade inflammatory diseases [11, 12].

Recent, albeit limited, studies have attempted to use SCFAs therapeutically in both animal and cell models of kidney injuries, such as ischemia-reperfusion-induced acute kidney injury (AKI) [13], contrast-induced nephropathy [14], and gentamicin-induced nephrotoxicity [15]. However, it is unclear whether exogenous SCFAs may directly prevent and ameliorate T2D-induced DN and how SCFAs may regulate this process. In this study, we first evaluated the effect of the three main SCFAs (acetate, propionate, and butyrate) on high-fat diet (HFD) and streptozotocin- (STZ-) induced T2D and DN mouse models *in vivo*. Next, we evaluated the effect of SCFAs on high glucose-induced oxidative stress and NF-*κ*B signaling in mouse glomerular mesangial cells (GMCs) *in vitro*. Finally, we investigated whether GPR43-*β*-arrestin-2 signaling could be involved in these positive effects of SCFAs by transfection with a GPR43 overexpressing vector or siRNA-GPR43. Our present study and findings may provide new insights on the role of microbiota metabolites in the intervention of DN.

## 2. Materials and Methods

### 2.1. Animal Model

Eight-week-old male C57BL/6 mice were purchased from the Biotechnology Corporation of Dashuo (Chengdu, China). All procedures were in accordance with the guidelines of the Institutional Animal Care and Use Committee of the Southwest Medical University.

At 10 weeks of age, all mice were randomly allocated to two groups: a control group (NC group, *n* = 8) and a T2D group (*n* = 32). The NC group received a normal diet until the end of experiments whereas the T2D mouse group was given a HFD (40% kcal fat; Biotechnology Corporation of Dashuo) for 8 weeks, and then diabetes was induced by intraperitoneal injection of single low-dose (40 mg/kg) STZ (Sigma-Aldrich, St Louis, MO, USA) dissolved in 0.05 M sodium citrate buffer (pH 4.5), followed by continued HFD feeding for an additional 12 weeks. Random blood glucose (RBG) levels of ≥16.7 mmol/L (mM) lasting 3 days were confirmed as being “diabetic.” At the moment of an STZ intraperitoneal injection, the T2D mice were further randomly classified into four groups (*n* = 8/group) with an equal average initial body weight: (1) acetate (Ac group): T2D mice treated with an intraperitoneal injection (100 mg/(kg·48 h) of sodium acetate (Sigma-Aldrich) for 12 weeks; (2) propionate (Pr group): T2D mice were treated with sodium propionate (Sigma-Aldrich) at a similar dose and frequency to those used for the Ac group; (3) butyrate (But group): T2D mice received the same dose and frequency of sodium butyrate (Sigma); and (4) T2D control group (T2D group): T2D mice treated with an intraperitoneal injection of phosphate-buffered saline (PBS) solution at the same volume and frequency. Meanwhile, the NC groups also received injections of equivalent volumes and frequency of PBS buffer. All mice were weighed, and blood samples from tail veins and urine specimens were collected every two weeks. At the 20th week of experiments, all mice were sacrificed and fasting heart blood collected; kidneys were used for pathology, western blotting, and qRT-PCR.

### 2.2. Biochemical Measurements

RBG and fasting blood glucose (FBG) levels were measured by Accu-Chek (Roche Diagnostics GmbH, Mannheim, Germany). Random urine albumin-creatinine ratios (ACR) were calculated every two weeks according to manufacturers' procedures as outlined in kits (Afinion™ ACR; Axis-Shield PoC AS, Oslo, Norway). Fasting insulin (FINS) levels were assayed by insulin enzyme-linked immunosorbent assay (ELISA) detection kit (Alpco, Salem, NH, USA), and HOMA-IR values were calculated according to a formula. Blood urea nitrogen (BUN), serum creatinine (SCr), serum cystatin C, total cholesterol (TC), triglyceride (TG), and low-density lipoprotein-cholesterol (LDL-C) levels were measured by an automatic biochemistry analyzer (Hitachi 7150, Hitachi Group, Tokyo, Japan).

### 2.3. Renal Histology

Kidneys from mice were fixed in 4% paraformaldehyde and embedded in paraffin, and 4 *μ*m sections were cut. Sections were stained with hematoxylin and eosin (H&E), periodic acid-Schiff (PAS), and Masson's trichrome staining. For each mouse, images of six different fields of view were evaluated under ×400 magnification by light microscopy (Leica, Wetzlar, Germany). The numbers of glomeruli with mesangial expansion and vasodilation were counted according to previously established methods [16]. The number of grid points on the mesangial matrix (PAS-stained glomerulus-positive areas) was divided by the total number of points in each glomerulus to calculate the relative mesangial matrix area as a percentage of the total grid of the glomerulus. Masson's trichrome-stained tissue images and relative fibrotic areas were evaluated by Image-Pro Plus 6.0 software. The histopathologist was blind to the treatment groups when analyses were made.

### 2.4. Immunohistochemistry Staining

Sections were incubated with the following primary antibodies: anti-GPR43 (rabbit polyclonal antibody; 1 : 100 dilution; Santa Cruz Biotechnology, Santa Cruz, CA, USA), anti-*β*-arrestin-2 (rabbit polyclonal antibody; 1 : 100 dilution; Santa Cruz Biotechnology), anti-NF-*κ*Bp65 (goat polyclonal antibody; 1 : 100 dilution; Santa Cruz Biotechnology), and anti-MCP-1 (rabbit monoclonal antibody; 1 : 100 dilution; CST, Danvers, MA, USA) overnight at 4°C. After sections were washed with PBS, they were incubated with horseradish peroxidase (HRP) or fluorescein isothiocyanate fluorescent dye-conjugated secondary antibodies (1 : 200 dilution; Beijing Biosynthesis Biotechnology, Beijing China) for 2 h at room temperature. For visualizing the signals of immunohistochemistry, sections were treated with peroxidase substrate 3,3-diaminobenzidine and counterstained with hematoxylin. Positive staining areas were evaluated by Image-Pro Plus 6.0 software.

### 2.5. Cell Culture, Treatment, and Viability Assay

Mouse glomerular mesangial cells (SV-40 MES 13) were obtained from the China Center for Type Culture Collection and cultured in Dulbecco's modified Eagle's medium (Gibco, Waltham, MA, USA) containing 5.6 mM glucose and 10% fetal bovine serum (Gibco) at 37°C and 5% CO_2_.

Initially, to determine proper concentrations of each SCFA and GPR43 agonist, cells were randomly divided and the following treatments were applied: (1) Ac at 0.1, 1, 10, and 100 mM concentrations; (2) Pr at 0.1, 1, 10, and 100 mM concentrations; (3) But at 0.05, 0.5, 5, and 50 mM concentrations; and (4) a phenylacetamide compound (Merck Millipore, Burlington, MA, USA) that acted as an allosteric agonist of GPR43 at 0.01 0.1, 1, and 10 *μ*mol/L (*μ*M) concentrations. GMCs were seeded in 96-well plates at concentrations of 1 × 10^5^ cells/mL to 50% confluence in DMEM complete growth medium, followed by treatments respectively supplemented with different concentrations of SCFAs and GPR43 agonist as described above for 24 h in full media. The viability of the cells was determined by a combination method including MTT analysis. MTT experiments were performed in 5 biological replicates. According to MTT results and literature reviews, 10 mM acetate, 10 mM propionate, 5 mM butyrate, and 1 *μ*M GPR43 agonist were used in the *in vitro* study.

### 2.6. GPR43 Overexpression Vector Construction and Transfection

Open reading frames of the mouse *GPR43* (Gene ID: 233079) gene were amplified by PCR and inserted in a pCD513B-1 plasmid (Public Protein/Plasmid Library) to construct a pCD513B-1-GPR43 expression vector (CMV promoter). PCR and restriction enzyme digestion were used to confirm a successfully constructed recombinant GPR43 overexpression vector. Transfection was done by a Lipofectamine®3000 RNAiMax reagent (Invitrogen, Karlsruhe, Germany) following the manufacturer's instructions.

### 2.7. Small Interfering RNA Transfection

siRNA targeting *GPR43* (sense: 5′-CCAGCCTGGATCCATTATT-3′, antisense: 5′-AAUAAUGGAUCCAGGCUGG-3′) or control siRNA (sense: 5′-UUCUCCGAACGUGUCACGU-3′; antisense: 5′-ACGUGACACGUUCGGAGAA-3′) was synthesized by Ribo Biotech (Guangzhou, China). Transfections were performed using the Lipofectamine® 3000 RNAiMax reagent (Invitrogen, Karlsruhe, Germany) following the manufacturer's instructions. Experiments were performed with these cells at 24 h posttransfection.

### 2.8. Detection of ROS, MDA, and SOD

After incubation with different compounds as described above, intracellular production of ROS was measured using an ROS assay kit (Beyotime, Haimen, China); the contents of MDA and total superoxide dismutase (SOD) were determined using a Lipid Peroxidation MDA Assay Kit (Beyotime) and a Total Superoxide Dismutase Assay Kit with WST-8 (Beyotime) according to the manufacturer's instructions. Values were expressed as the mean absorbance normalized to a percentage of the normal control.

### 2.9. Western Blotting

Total protein was extracted using a protein extraction kit (Kaiji, Shanghai, China), which contains a protease inhibitor and phosphatase inhibitor. Protein concentrations were assessed by using a BCA Protein Assay Kit (Bioworld Technology, USA). The OD value was measured at a wavelength of 562 nm, and the standard curve (*r* > 0.99) calculated the protein sample concentration. Proteins were separated by sodium dodecyl sulfate-polyacrylamide gel electrophoresis and transferred to a polyvinylidene difluoride membrane (Merck Millipore). Immunoblotting was performed using anti-GPR43 (rabbit polyclonal antibody; 1 : 1000 dilution; Santa Cruz Biotechnology), anti-*β*-arrestin-2 (rabbit polyclonal antibody; 1 : 1000 dilution; Santa Cruz Biotechnology), anti-I-*κ*B*α* antibody (mouse monoclonal antibody; 1 : 1000; CST, number 4814), anti-p-I-*κ*B*α* antibody (ser32/36; mouse monoclonal antibody; 1 : 1000; CST; number 9246), anti-NF-*κ*Bp65 antibody (rabbit polyclonal antibody; 1 : 1000; Beyotime; number AF0246), anti-p-NF-*κ*Bp65 antibody (ser536; mouse monoclonal antibody; 1 : 2000; CST; number 13346), anti-MCP-1 (rabbit monoclonal antibody; 1 : 100 dilution; CST), anti-IL-1*β* (rabbit polyclonal antibody; 1 : 800 dilution; CST), and anti-GAPDH antibody (mouse; 1 : 800 dilution; Beyotime) overnight at 4°C. The second antibodies of GPR43, *β*-arrestin-2, NF-*κ*Bp65, MCP-1, and IL-1*β* (1 : 2000, anti-rabbit) and I-*κ*B*α*, p-I-*κ*B*α*, p-NF-*κ*Bp65, and GAPDH (1 : 2000, anti-mouse) were obtained from the Beyotime Institute of Biotechnology, Shanghai, China. The proteins were detected with HRP chemiluminescence reagent (Millipore, USA), and images were captured with the UVP imaging system (Bio-Rad, USA). Quantity One software was used for the analysis of bands.

### 2.10. Quantitative Real-Time PCR Analysis

Total RNA was isolated from the kidney and GMCs using an RNA extraction kit (ComWin Biotech, Beijing, China). The isolated RNA was subjected to reverse transcription using a PrimeScript RT Reagent Kit (TaKaRa, Kusatsu, Japan). The synthesized cDNA was used as a template for quantitative PCR analysis. The housekeeping gene, *β*-actin, was quantified as an internal RNA control. Quantitative RT-PCR was performed on a 7900HT Fast Real-Time PCR System (Thermo Fisher Scientific, Waltham, MA, USA). The primer sequences for all studied genes are listed in Table 1. The thermal cycling program used was as follows: an initial step at 95°C for 10 min, followed by 40 cycles of denaturation at 95°C for 15 s, annealing at 60°C for 34 s, and extension at 72°C for 15 s. The melting curve of each PCR product was obtained by continuous fluorescence monitoring at a temperature gradient ramp from 60 to 95°C. Quantitative PCR reactions were performed in triplicate to remove any outliers. The relative changes in gene expression were analyzed by the 2-*ΔΔ*CT method.

### 2.11. ELISA Assay

MCP-1 and IL-1*β* protein levels in cell culture supernatants were determined using commercially available MCP-1 and IL-1*β* ELISA kits (Neobioscience, Shanghai, China) according to the manufacturer's protocols. MCP-1 and IL-1*β* protein levels were determined by comparing the samples with a standard curve generated using the kit.

### 2.12. Statistics

All data were obtained from at least five independent experiments and were expressed as the mean ± standard deviation (SD). Between-group comparisons were analyzed using one-way analysis of variance (ANOVA), followed by post hoc Tukey's correction test for multiple comparisons (SPSS 20.0 software). *p* < 0.05 was considered significant.

## 3. Results

### 3.1. SCFAs Ameliorated Hyperglycemia and Insulin Resistance of Experimental T2D

**Figure 1:**
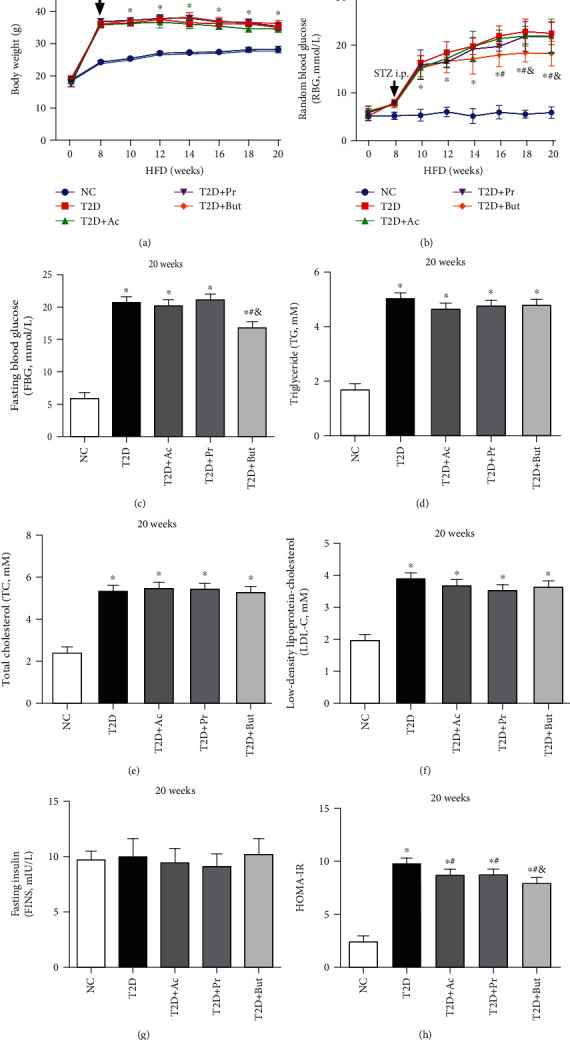
SCFAs ameliorated hyperglycemia and insulin resistance of experimental *T2D*. Mice were subjected to a high-fat diet (HFD) for 8 weeks, intraperitoneally (i.p.) injected with STZ, and then treated with three main SCFAs, acetate (Ac), propionate (Pr), and butyrate (But), for 12 weeks. Body weight (BW) (a) and random blood glucose (RBG) (b) were measured every 2 weeks; fasting blood glucose (FBG) (c), total cholesterol (TC) (d), total glyceride (TG) (e), low-density lipoprotein-cholesterol (LDL-C) (f), fasting insulin (FINS) (g), and homeostatic model assessment of insulin resistance (HOMA-IR) (h) values were measured at the 20th week of the experiment before sacrifice. ^∗^*p* < 0.05 compared with the NC group; ^#^*p* < 0.05 compared with the T2D group; ^&^*p* < 0.05 compared with the Ac or Pr group.

In the current study, we investigated the role of SCFAs in a nongenetic rodent model of T2D, HFD/STZ mice. In this model, overt hyperglycemia results from a combination of insulin resistance induced by HFD feeding and defects in insulin secretion induced by single low-dose (40 mg/kg) STZ treatment. To examine the effects of SCFAs on glycolipid metabolism in the development of obesity and insulin resistance, body weight (BW, Figure 1(a)) and RBG (Figure 1(b)) levels were assessed at baseline and from 8 to 20 weeks. After 8 weeks, compared with the NC group, significant changes in BW and RBG levels in T2D mice were noted. This pattern was also seen for FBG (Figure 1(c)), blood lipid spectrum (TC, TG, and LDL-C; Figures 1(d)–1(f)), and FINS (Figure 1(g)) levels, suggesting that T2D models were successfully achieved. Next, we found that intraperitoneal injections of three main SCFAs for 12 weeks did not have a significant effect on BW, FINS, and the blood lipid spectrum in experimental T2D mice. However, when supplemented with SCFAs, especially butyrate, RBG and FBG levels were partially reversed (Figures 1(a)–1(c)). Finally, SCFA-supplemented mice showed significantly lower homeostatic model assessment of HOMA-IR (Figure 1(h)) than T2D controls and enhanced insulin sensitivity, without affecting the FINS (Figure 1(g)). Collectively, our observations indicated that exogenous SCFAs did not significantly affect obesity, FINS, and lipid metabolism; however, SCFAs, especially butyrate, improved insulin resistance and protected mice from experimentally induced T2D.

### 3.2. SCFAs Prevented Renal Dysfunction in Experimental T2D

**Figure 2:**
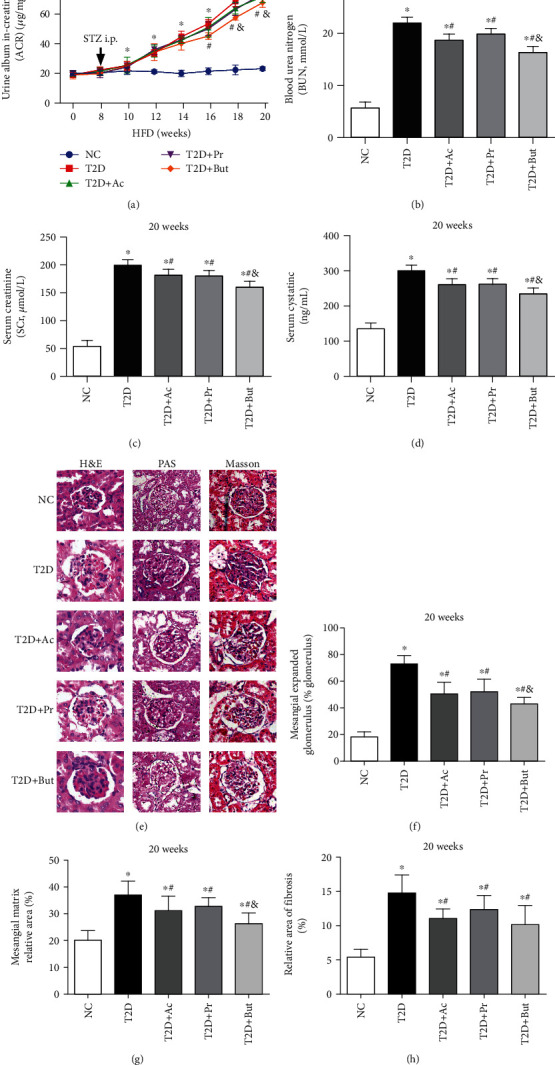
SCFAs prevented the renal dysfunction and kidney injury. Urine ACR (a) of T2D mice were measured every 2 weeks, and blood urea nitrogen (BUN) (b), serum creatinine (SCr) (c), and serum cystatin C (d) were assayed at the 20th week of the experiment. Histopathological examination of renal tissues was by H&E, PAS, and Masson's trichrome staining (400x) (e). Mesangial expansion (f), glomerular tuft (g), and the accumulation of collagen (h) were measured. Ac: acetate; Pr: propionate; But: butyrate; ^∗^*p* < 0.05 compared with the NC group; ^#^*p* < 0.05 compared with the T2D group; ^&^*p* < 0.05 compared with the Ac or Pr group.

To assess whether SCFAs were nephroprotective *in vivo*, random ACR, an important feature of kidney injury in DN, were measured every two weeks. We found that SCFA treatment, especially butyrate, resulted in lowering the levels of urine ACR (Figure 2(a)) and in lowering serum urea (Figure 2(b)), creatinine (Figure 2(c)), and cystatin C (Figure 2(d)) levels, markers of the severity of renal dysfunction in DN. Histopathological examination of renal tissues by H&E, PAS stains, and Masson's trichrome (Figure 2(e)) revealed that mesangial expansion (Figure 2(f)), the glomerular tuft (Figure 2(g)), and the accumulation of collagen (Figure 2(h)) were substantially elevated in the T2D group when compared to the NC group. Notably, these histomorphometric changes were significantly attenuated by treatment with SCFAs, especially butyrate. These results indicated that T2D-induced renal histomorphometric changes and renal dysfunction were effectively ameliorated by SCFAs.

### 3.3. SCFAs Inhibited T2D-Induced Renal NF-*κ*B Activation and Regulated GPR43-*β*-Arrestin-2 Signaling

**Figure 3:**
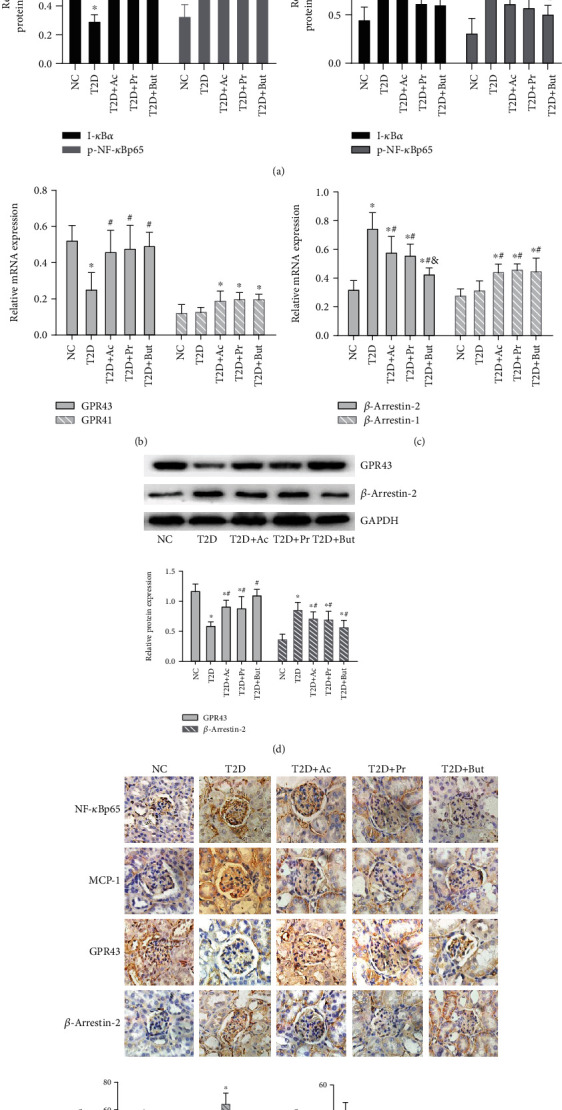
SCFAs inhibited T2D-induced NF-*κ*B activation and regulate GPR43-*β*-arrestin-2 signaling. (a) Western blotting revealed the expression of I-*κ*B*α*, p-NF-*κ*Bp65, MCP-1, and IL-1*β* in T2D kidney tissue after SCFA treatment. (b, c) qRT-PCR of GPR43, GPR41, *β*-arrestin-2, and *β*-arrestin-1 in kidney tissue after SCFA treatment. (d) Western blotting-based assays for the expression of GPR43 and *β*-arrestin-2 in kidney tissue after SCFA treatment. (e) Immunohistochemistry- (400x) based assays for the expression of p-NF-*κ*Bp65, MCP-1, GPR43, and *β*-arrestin-2 after SCFA treatment. Ac: acetate; Pr: propionate; But: butyrate; ^∗^*p* < 0.05 compared with the NC group; ^#^*p* < 0.05 compared with the T2D group; ^&^*p* < 0.05 compared with the Ac or Pr group.

We searched for potential target genes of SCFAs in the treatment of DN. According to the literature, a possible candidate was NF-*κ*Bp65, which forms a key part in the multiple signal transduction of chronic inflammation in the pathogenesis of DN. Consistent with prior reports, western blotting showed that the protective effects of SCFAs, especially butyrate, were associated with the increased expression of I-*κ*B*α*, which inhibits the phosphorylation and nuclear translocation of NF-*κ*Bp65, and inhibited the downstream inflammatory cytokine, MCP-1, and IL-1*β* expression (Figure 3(a)). In order to determine if there was a relationship between GPCRs and ***β***-arrestins in established experimental DN mice, we measured mRNA levels of GPR43, GPR41, ***β***-arrestin-1, and ***β***-arrestin-2 by qRT-PCR. Compared with the NC group, mRNA expression of GPR43 was inhibited, but ***β***-arrestin-2 were upregulated in the kidneys of T2D mice. However, no obvious response was found for GPR41 and ***β***-arrestin-1; SCFAs induced the mRNA expression of GPR43 (Figure 3(b)) but inhibited that of ***β***-arrestin-2 (Figure 3(c)) simultaneously. Furthermore, both western blotting (Figure 3(d)) and immunohistochemistry (Figure 3(e)) confirmed that SCFAs reversed the T2D-induced downregulation of GPR43 and upregulation of ***β***-arrestin-2 and inhibited p-NF-*κ*Bp65 and MCP-1 expression, suggesting that SCFAs have a protective effect on DN by regulating GPR43-***β***-arrestin-2 and inhibiting the activation of NF-*κ*B signaling.

### 3.4. SCFAs Partially Reversed High Glucose-Induced Oxidative Stress and NF-*κ*B Signaling Activation *In Vitro*

**Figure 4:**
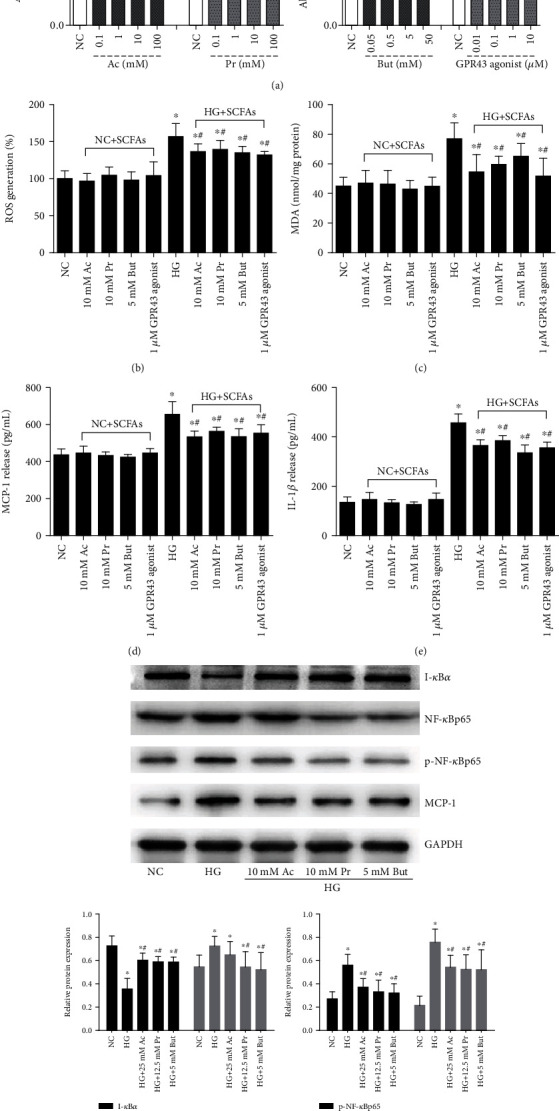
SCFA treatment partially inhibited oxidative stress and NF-*κ*B activation in high glucose-induced GMCs. (a) The effects of a concentration range of SCFAs or GPR43 agonist on GMC proliferation were analyzed by MTT assay. GMCs were stimulated with 30 mM high glucose in the presence of the indicated concentration of SCFAs or GPR43 agonist for 24 h. ROS (b), MDA (c), MCP-1 (d), and IL-1*β* (e) in the cell culture supernatant were evaluated by kit. The protein expression of I-*κ*B*α*, NF-*κ*Bp65, p-NF-*κ*Bp65, and MCP-1 was assayed by western blotting (f). Ac: acetate group; Pr: propionate group; But: butyrate group; ^∗^*p* < 0.05 compared with the NC group; ^#^*p* < 0.05 compared with the HG group.

**Figure 5:**
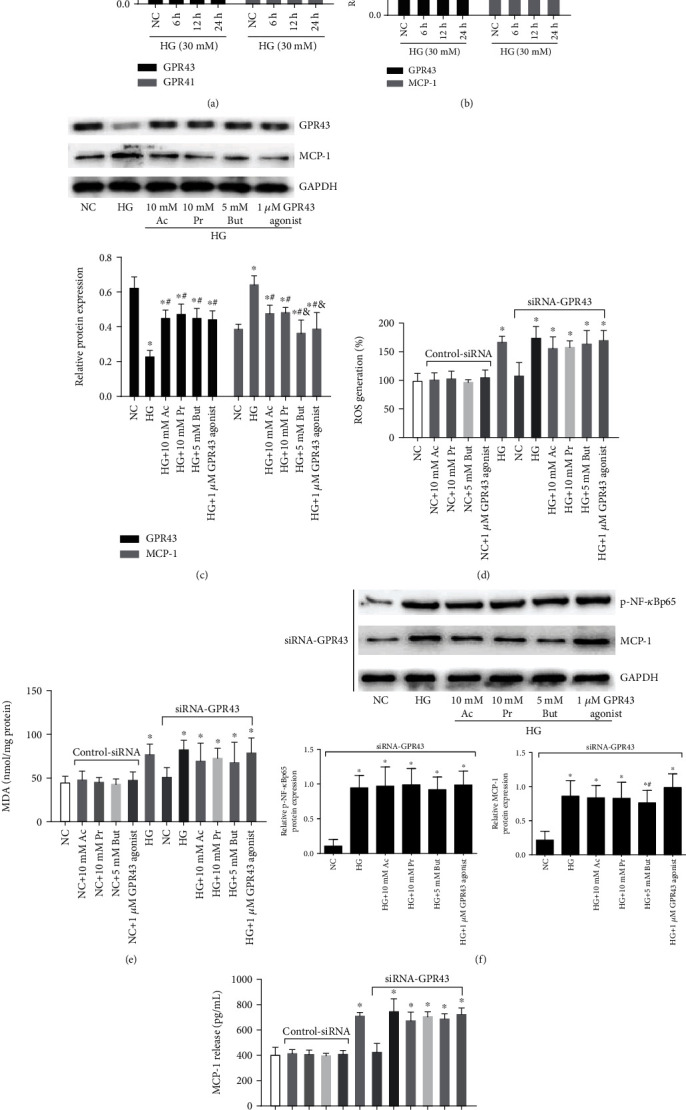
SCFA-mediated antioxidant and anti-inflammatory effects were partly reversed by siRNA-GPR43. qRT-PCR was performed to detect GPR43 and GPR41 mRNA levels. (b) Western blotting-based assay for the expression of GPR43 and MCP-1 after a 30 mM high glucose challenge for 6, 12, and 24 h. (c) The effects of indicated concentrations of SCFAs or a GPR43 agonist on GPR43 and MCP-1 expression were analyzed by western blotting. Following 30 mM high glucose for 24 h, SCFAs or a GPR43 agonist-ameliorated ROS (d), MDA (e), NF-*κ*B signal (g), and MCP-1 (f) were significantly reversed by siRNA-GPR43. Ac: acetate; Pr: propionate; But: butyrate; ^∗^*p* < 0.05 compared with the NC group; ^#^*p* < 0.05 compared with the HG group. ^&^*p* < 0.05 compared with the Ac or Pr group.

Next, we explored whether oxidative stress and NF-*κ*B signaling could be causally involved in the renal protective effect of SCFAs *in vitro*. Treating GMCs for 24 h with 0.1-10 mM concentration range of acetate or propionate, 0.05-5 mM butyrate, or 0.1-1 *μ*M GPR43 agonist promoted GMC proliferation in a dose-dependent manner; however, higher concentrations of these SCFAs or GPR43 agonist inhibited cell viability (Figure 4(a)). According to the MTT assay and literature review, 10 mM acetate, 10 mM propionate, 5 mM butyrate, and 1 *μ*M GPR43 agonist were used as intervention reagents *in vitro*; 30 mM high glucose was then used as a stimulating factor, which induced abnormal levels of oxidative stress-relevant molecules such as ROS (Figure 5(b)) and MDA (Figure 5(c)), as well as the release of MCP-1 (Figure 5(d)) and IL-1*β* (Figure 5(e)). We found that these high glucose-induced abnormalities were significantly abolished by SCFAs or the GPR43 agonist, and these intervention reagents have no significant effect on oxidative stress and inflammation. In line with *in vivo* results, western blotting showed that the high glucose-induced phosphorylation of NF-*κ*Bp65 and MCP-1 protein level was significantly decreased by these SCFAs or a GPR43 agonist (Figure 5(f)), suggesting that either SCFAs or a GPR43 agonist at a certain concentration range inhibited high glucose-induced oxidative stress and NF-*κ*B activation in GMCs.

### 3.5. SCFA-Mediated Antioxidant and Anti-Inflammatory Effects Were Partially Reversed by siRNA-GPR43

To determine whether GPR43 and GPR41 are regulated by high glucose *in vitro*, we firstly detected GPR43 and GPR41 mRNA in GMCs by qRT-PCR. Compared with the NC group, the relative GPR43 mRNA expression gradually decreased after treatment with 30 mM glucose from 6 h to 24 h; however, a change in the GPR41 level was not found with high glucose treatment (Figure 5(a)), in line with *in vivo* results. Western blotting (Figure 5(b)) confirmed that GPR43 expression in GMCs was gradually decreased by 30 mM glucose in a time-dependent manner, accompanied by the high expression of MCP-1, revealing an intrinsic relationship between GPR43 and chronic inflammation in GMCs. However, the high glucose-inhibited GPR43 as well as high glucose-induced MCP-1 were abolished by SCFAs or the GPR43 agonist (Figure 5(c)). To find out whether GPR43 mediate the role of SCFAs, siRNAs were constructed to silence the GPR43 gene in GMCs. The results showed that the inhibition of ROS (Figure 5(d)) and MDA (Figure 5(e)) by SCFAs or the GPR43 agonist was significantly reversed by siRNA-GPR43. Furthermore, SCFAs or the GPR43 agonist-inhibited NF-*κ*B activation, MCP-1 expression (Figure 5(f)), and MCP-1 release (Figure 5(g)) were also abolished by siRNA-GPR43, suggesting that GPR43 was partially involved in SCFA-mediated antioxidant and anti-inflammatory effects.

### 3.6. SCFA-Mediated Beneficial Effects Were Significantly Facilitated by GPR43 Overexpression

**Figure 6:**
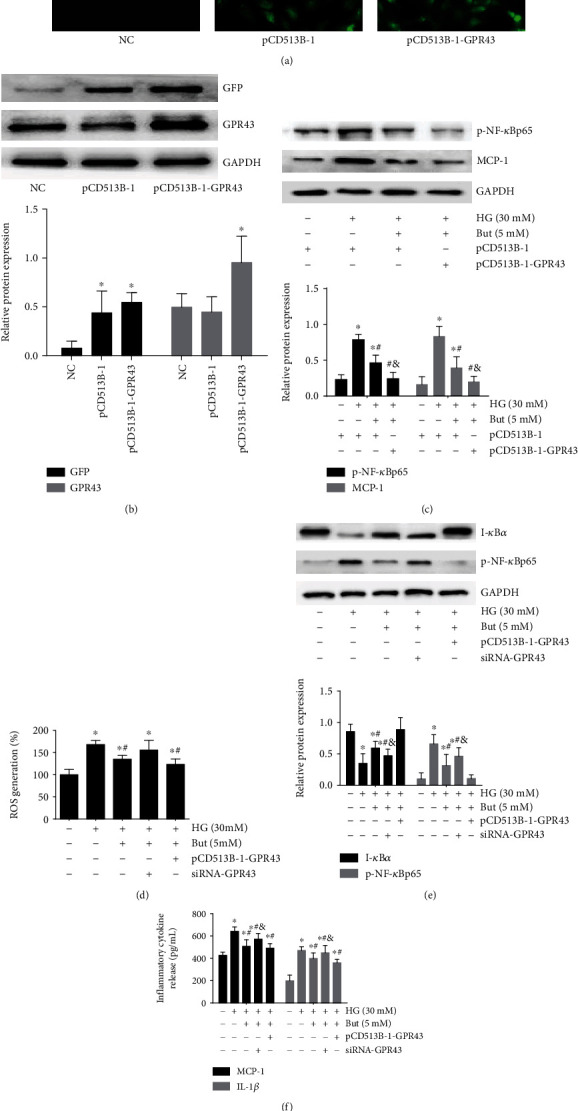
SCFA-mediated antioxidant and anti-inflammatory effects were significantly facilitated by GPR43 overexpression. Plasmids expressing GPR43 with pCD513B-1 (an N-terminal GFP tag) and pCD513B-1 control plasmid that expresses GFP but cannot overexpress GPR43 were constructed to determine the effect of overexpressed GPR43 and GFP protein in GMCs according to fluorescence images (200x) (a) and western blotting (b). Following 30 mM high glucose for 24 h, 5 mM But-mediated inhibition of p-NF-*κ*Bp65 and MCP-1 protein expression (c) was significantly facilitated by GPR43 overexpression. The But-mediated inhibition of ROS (d) and p-NF-*κ*Bp65 (e) and MCP-1 and IL-1*β* release (f) were reversed by siRNA-GPR43 but facilitated by GPR43 overexpression. But butyrate: ^∗^*p* < 0.05 compared with the NC group, ^#^*p* < 0.05 compared with the HG group, and ^&^*p* < 0.05 compared with the But group.

To assess the involvement of GPR43 in the inhibition of NF-*κ*B signaling, plasmids overexpressing mouse GPR43 with pCD513B-1 (a GFP tag) or the pCD513B-1 control plasmid was constructed to determine the effects of overexpressed GPR43 on SCFA-mediated benefits. GMCs showed green fluorescence (Figure 6(a)), suggesting plasmid transfection was successful and the pCD513B-1-GPR43 fusion protein was expressed. Western blotting showed that the GPR43 were overexpressed in GMCs after transfection (Figure 6(b)). Since the most dramatic changes were observed in the butyrate intervention group, we only measured butyrate-mediated benefits in the next study. The results showed that butyrate-inhibited phosphorylation of NF-*κ*Bp65 and MCP-1 expression (Figure 6(c)) was enhanced by overexpressed GPR43. Furthermore, the butyrate-inhibited ROS (Figure 6(d)) and NF-*κ*B components (Figure 6(e)) and MCP-1 and IL-1*β* release (Figure 6(f)) were also reversed by siRNA-GPR43 but were facilitated by GPR43 overexpression. Taken together, these data indicated that the inhibition of oxidative stress and NF-*κ*B signaling by SCFAs was partially dependent on GPR43.

### 3.7. The Interaction of *β*-Arrestin-2 and I-*κ*B*α* Was Induced by SCFAs via GPR43

**Figure 7:**
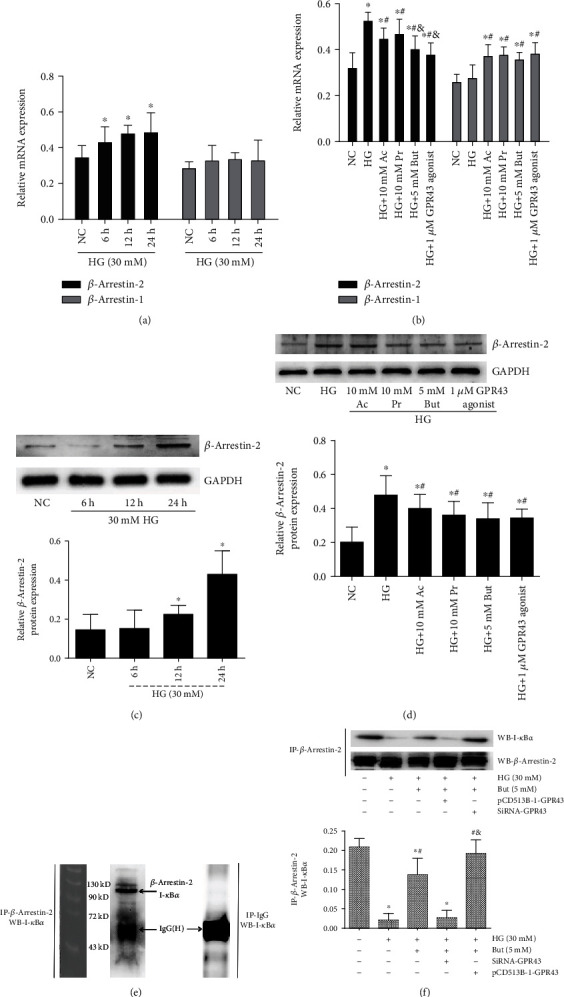
Interaction between *β*-arrestin-2 and I-*κ*B*α* was induced by SCFAs via GPR43. (a) GMCs were treated with 30 mM high glucose for 6, 12, and 24 h. RT-PCR was performed to detect *β*-arrestin-2 and *β*-arrestin-1 mRNA levels. (b) The effects of indicated concentrations of SCFAs or an GPR43 agonist on *β*-arrestin-2 and *β*-arrestin-1 expression were analyzed by RT-PCR. (c) Western blot assay for the expression of *β*-arrestin-2 after 30 mM high glucose challenge for 6, 12, and 24 h. (d) High glucose-induced *β*-arrestin-2 expression was significantly reversed by SCFAs or GPR43 agonist. (e) The interaction between *β*-arrestin-2 and I-*κ*B*α* under physiological conditions was detected by immunoprecipitation (IP) with anti-*β*-arrestin-2 antibody or normal mouse IgG antibody (negative control), followed by western blotting with an anti-I-*κ*B*α* antibody. *β*-Arrestin-2 was conjugated with I-*κ*B*α* in *vitro*. (f) The interaction between *β*-arrestin-2 and I-*κ*B*α* was decreased by 30 mM high glucose but was reversed by 5 mM butyrate. And these butyrate-mediated effects were significantly reversed by siRNA-GPR43 but were facilitated by overexpressed GPR43. IgG-H marks the IgG heavy chain. But butyrate: ^∗^*p* < 0.05 compared with the NC group, ^#^*p* < 0.05 compared with the HG group, and ^&^*p* < 0.05 compared with the HG+But group. Ac: acetate; Pr: propionate.

To investigate the underlying mechanism by which GPR43-*β*-arrestin-2 signaling was involved in SCFA-inhibited NF-*κ*B signaling, we first performed experiments to determine whether *β*-arrestins respond to high glucose and SCFAs. qRT-PCR showed that compared with *β*-arrestin-1, 30 mM high glucose induced the mRNA expression of *β*-arrestin-2 in a time-dependent manner (Figure 7(a)). SCFAs, especially butyrate, or the GPR43 agonist, inhibited high glucose-induced *β*-arrestin-2 expression (Figure 7(b)). Consistent with mRNA expression, western blotting confirmed that the *β*-arrestin-2 expression was induced by high glucose in a time-dependent manner (Figure 7(c)), but this trend was reversed by SCFAs or the GPR43 agonist (Figure 7(d)). Next, cell extracts were prepared and subjected to immunoprecipitation using anti-*β*-arrestin-2 antibody, and I-*κ*B*α* coimmunoprecipitation was detected by immunoblotting with anti-I-*κ*B*α* antibodies. As shown in Figure 7(e), *β*-arrestin-2 antibodies immunoprecipitated a polypeptide of about 100 kDa that was recognized by the I-*κ*B*α*-specific antibody (Figure 7(e)), indicating that *β*-arrestin-2 and I-*κ*B*α* were able to form a complex on endogenously expressed proteins in GMCs. Interestingly, the results also revealed that the interaction between *β*-arrestin-2 and I-*κ*B*α* was decreased by 30 mM high glucose, but this effect was reversed by 5 mM butyrate. To investigate the underlying mechanism, GPR43 overexpression plasmids and siRNA-GPR43 were transfected into GMCs; the coimmunoprecipitation showed that butyrate-induced interaction between *β*-arrestin-2 and I-*κ*B*α* was significantly reversed by siRNA-GPR43 but was facilitated by GPR43 overexpression (Figure 7(f)), suggesting that butyrate inhibited NF-*κ*B signaling by GPR43-mediated interaction between *β*-arrestin-2 and I-*κ*B*α*.

## 4. Discussion

SCFAs were identified as a link between gut microbiota and T2D: a number of studies in the last decade have shown that increased intake of dietary fiber and dietary supplementation with butyrate prevented or treated diet-induced weight gain, insulin resistance, and its related metabolic comorbidities [17, 18]. Recently, the effect of oral butyrate administration on blood HbA1c, inflammatory cytokines, and lipopolysaccharide (LPS) was demonstrated in db/db mice by restoring the composition of gut microbiota and preserving the integrity of the gut epithelial barrier [19]. SCFAs have been shown to modulate intestinal hormones (such as glucagon-like peptide 1 and peptide tyrosine tyrosine) and to affect intestinal permeability, satiety, gastric emptying, and food intake [20, 21]. Recent studies have revealed that SCFAs regulate the inflammatory response and metabolic homeostasis [22–24]. However, a study of external SCFA administration on glucose and lipid metabolism in HFD and STZ-induced T2D mice is lacking. In the present study, we found that the intraperitoneal injection of three main SCFAs had no significant effect on BW, FINS, and the blood lipid spectrum of T2D mice; however, SCFA treatment, especially butyrate, resulted in decreased RBG, FBG, and HOMA-IR, suggesting a positive effect of SCFAs on insulin sensitivity and glucose homeostasis by a nongastrointestinal interventional manner. We speculate that SCFAs protect T2D mice by improving hepatic and peripheral insulin resistance as reported in the literatures above.

Inadequate levels of SCFAs are associated with kidney and cardiovascular disease [25–27]. Importantly, the latest studies found that an intraperitoneal injection of sodium butyrate or its oral administration can improve renal dysfunction and inhibit renal oxidative stress, inflammation, and fibrosis in the STZ-induced T1D mouse [28, 29]. SCFAs exert their anti-inflammatory effects partly via preventing the proteasomal degradation of the NF-*κ*B inhibitor, I-*κ*B*α*, both in intestinal and in extraintestinal environments [16, 30]. Here, we found that elevated ACR, serum urea, creatinine, and cystatin C, markers of the severity of renal dysfunction in DN, were decreased by SCFAs. In addition, mesangial expansion, the glomerular tuft, the accumulation of collagen, and the activation of NF-*κ*B in diabetic renal tissues were significantly attenuated by SCFAs, especially butyrate. In *in vitro* studies, the inhibitory effects of SCFAs on oxidative stress and inflammation have been reported in porcine kidney cells and human renal cortical and tubular epithelial cells [31, 32]. GMCs are vulnerable to external stimulation, such as high glucose environment, which induces proliferation, hypertrophy, and extracellular matrix accumulation, as well as consequent renal fibrosis. These pathophysiological changes have been recognized as major events in the progression of DN; therefore, in this study, GMCs were used as a representative cell type [33, 34]. Although limited by cell type, our study found that the protective effects of SCFAs were associated with the inhibition of ROS and MDA and suppressed the degradation of I-*κ*B*α* and the phosphorylation of NF-*κ*Bp65 in high glucose-induced GMCs. Taking into account all of these recent studies and the present results, SCFAs, especially butyrate, have shown positive effects on diabetic kidney injury, inhibition of oxidative stress, and NF-*κ*B signaling and may be potential therapeutic agents in the prevention and treatment of DN.

However, unlike the positive effects of SCFAs on AKI models that have been observed, the influence of SCFAs on chronic kidney disease (CKD) seems to be more controversial: either protective or causative effects exist. Park et al. [35] showed that oral administration of SCFAs induced chronically increasing doses of SCFAs to higher than physiological levels in mice and led to kidney hydronephrosis. Therefore, SCFAs were confirmed to play a dual role in the inflammatory system depending on the stimulus concentration [36]. Furthermore, although SCFAs are weak acids, too high concentrations or too frequent injections may stimulate the peritoneum to induce aseptic inflammation and ascites formation. Based on literature reports on *in vivo* experiments [13–15], our study indicated that a 100 mg/kg/48 h dose of SCFAs had positive and tolerable effects in T2D model mice. Previous studies *in vitro* have revealed that 25 mM acetate, 12 mM propionate, and 3.2 mM butyrate improved hypoxia-induced MitoSOX in renal tubular epithelial cells (HK-2 cells) [13]. In addition, 0.5-10 mM butyrate uppressed high glucose-stimulated TGF-*β*1 synthesis in HK-2 cells in a dose-dependent manner [37]. In line with the literature above, we showed that 10 mM acetate, 10 mM propionate, or 5 mM butyrate has protective effects on high glucose-induced oxidative stress and inflammation. It would seem contradictory that the SCFA pharmacological concentrations used herein or in the literature should be at least at the mM level to inhibit the expression of cytokines; however, the concentration of SCFAs in the peripheral circulation is very low (19-160 *μ*M), especially that of propionate and butyrate [38]. Therefore, low concentration of SCFAs would not be able to induce antioxidant and anti-inflammatory effects; however, if the concentration of SCFAs is elevated by exogenous supplementation, some tissues and cells may not be able to tolerate it [39]. These studies and our results suggest the importance of determining appropriate concentrations when testing the benefits of SCFAs in kidney disease and encourage further studies to identify the most appropriate interventional manner and related pharmacological concentration.

Finally, we intended to explore the molecular mechanism (s) by which SCFAs mediate antioxidant and anti-inflammatory effects. Compared with the GPR41 receptor, GPR43 is mostly involved in the regulation of immune function and inflammation [40–42]. Our previous study revealed that SCFAs or a GPR43 agonist markedly upregulated the expression of GPR43 inhibited by high glucose but diminished the expression of MCP-1 and IL-1*β* [43]. In the present study, we showed that compared with GPR43, GPR41 was rarely expressed in kidney tissues and in GMCs and did not show an obvious response to diabetes-relative stimulation; SCFAs reversed the downregulation of GPR43, along with inhibition of oxidative stress and NF-*κ*B signaling, indicating that GPR43 was critically involved in SCFA-mediated beneficial effects. Recently, several studies found that *β*-arrestin-2, which regulates desensitization, internalization, intracellular signaling, and recycling of GPCRs, directly binds to and blocks phosphorylation and degradation of I-*κ*B*α* and finally leads to the inhibition of NF-*κ*B activity [44, 45]. The most important finding presented here is that high glucose induced the expression of *β*-arrestin-2, but not *β*-arrestin-1, in a time-dependent manner, suggesting that *β*-arrestin-2 may represent a new target for an anti-inflammatory therapy, in response to SCFAs. Our study also revealed that I-*κ*B*α* coimmunoprecipitated with *β*-arrestin-2 under physiological conditions, but the interaction was attenuated by high glucose; furthermore, upon butyrate treatment, an increased amount of I-*κ*B*α* was associated with *β*-arrestin-2, illustrating that butyrate blocks phosphorylation and degradation of I-*κ*B*α* by inducing the interaction between *β*-arrestin-2 and I-*κ*B*α*, finally leading to the inhibition of NF-*κ*B signaling. Last but not least, the butyrate-induced positive effects described above were significantly inhibited by siRNA-GPR43 or facilitated by overexpressed GPR43. These results collectively suggest that the interaction between *β*-arrestin-2 and I-*κ*B*α* is induced by SCFAs via GPR43; GPR43-*β*-arrestin-2 signaling may be a new and promising target for DN.

It must be pointed out that some of the beneficial effects of butyrate administration were statistically significant in *in vivo* and *in vitro* experiments; however, SCFA treatment did not totally reverse T2D-induced renal dysfunction, and high glucose-induced oxidative stress and NF-*κ*B activation were not totally inhibited by SCFAs or a GPR43 agonist, suggesting the presence of other mechanisms, such as the inhibition of HDAC, which are involved in the crosstalk of SCFAs and the kidney in the prevention and treatment of DN [8, 28, 29]. Therefore, highly selective agonists and antagonists for receptors that sense SCFAs as well as tissue-specific GPR43 and/or *β*-arrestin-2 knockout or overexpressing mice are needed in future studies to elucidate the molecular mechanisms involved in SCFA-mediated benefits [46]. Finally, compared with other SCFAs, butyrate has shown a better therapeutic effect, but in relatively small absolute numbers; how to select the best efficient SCFA subtype, control the concentration, and avoid potential side effects are important challenges for future research.

## 5. Conclusion

**Figure 8:**
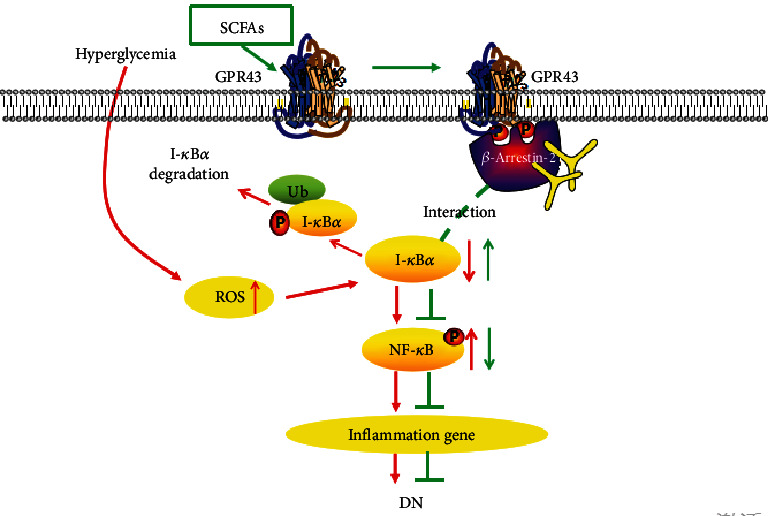
Overview on the effects of SCFAs on oxidative stress and NF-*κ*B activation in DN. High glucose induces the production of ROS and the polyubiquitination of phosphorylated I-*κ*B*α*, followed by NF-*κ*B activation and the expression of various inflammatory cytokines that are important factors in the development of DN (red arrows). However, SCFAs inhibit the oxidative stress and NF-*κ*B inflammatory signaling possibly via activating GPR43 and increasing the interaction between *β*-arrestin-2 and I-*κ*B*α* (green arrows), suggesting that SCFA-mediated GPR43-*β*-arrestin-2 signaling may be a novel and promising target for DN.

As summarized in Figure 8, our study provides compelling evidence that exogenous SCFAs, especially butyrate, ameliorate hyperglycemia and insulin resistance, improve renal function, ameliorate histopathological changes, and restore high glucose-induced inflammatory damage, which is attributable to the role GPR43-*β*-arrestin-2 signaling plays in buffering oxidative stress and blocking NF-*κ*B activation. These results from the current study establish a novel role of SCFAs in improvement of glucose metabolism and renal protection through GPR43-*β*-arrestin-2 machinery and may have implications for DN therapy.

## Figures and Tables

**Table 1:** Primer sequences for quantitative real-time PCR.

Gene	Forward sequence	Reverse sequence
GPR43	5′-GGTGGAGGCTGTGGTGTT-3′	5′-GCATAGAGGAGGCAGGATT-3′
GPR41	5′-CTCATCACCAGCTACTGCCG-3′	5′-AATTCAGGGTGCTGAGGAGC-3′
*β*-Arrestin-2	5′-CCATTGTGAAGGAGGGAG-3′	5′-GCATTAGGACGAAGGGTAG-3′
*β*-Arrestin-1	5′-ACCTTTGAGATCCCGCCAAA-3′	5′-CTTTCTGATGATAAGCCGCACA-3′
*β*-Actin	5′-ACCTCTATGCCAACACAGTG-3′	5′-GGACTCATCGTACTCCTGCT-3′

## Data Availability

The data used to support the findings of this study are available from the corresponding author upon request.
